# Predicting the risk of prostate cancer in asymptomatic men: a cohort study to develop and validate a novel algorithm

**DOI:** 10.3399/bjgp20X714137

**Published:** 2021-04-20

**Authors:** Julia Hippisley-Cox, Carol Coupland

**Affiliations:** Nuffield Department of Primary Care Health Sciences, University of Oxford, Oxford.; Division of Primary Care, University of Nottingham, Nottingham.

**Keywords:** cohort studies, primary health care, prostate cancer, prostate-specific antigen, risk prediction

## Abstract

**Background:**

Diagnosis of prostate cancer at an early stage can potentially identify tumours when intervention may improve treatment options and survival.

**Aim:**

To develop and validate an equation to predict absolute risk of prostate cancer in asymptomatic men with prostate specific antigen (PSA) tests in primary care.

**Design and setting:**

Cohort study using data from English general practices, held in the QResearch database.

**Method:**

Routine data were collected from 1098 QResearch English general practices linked to mortality, hospital, and cancer records for model development. Two separate sets of practices were used for validation. In total, there were 844 455 men aged 25–84 years with PSA tests recorded who were free of prostate cancer at baseline in the derivation cohort; the validation cohorts comprised 292 084 and 316 583 men. The primary outcome was incident prostate cancer. Cox proportional hazards models were used to derive 10-year risk equations. Measures of performance were determined in both validation cohorts.

**Results:**

There were 40 821 incident cases of prostate cancer in the derivation cohort. The risk equation included PSA level, age, deprivation, ethnicity, smoking status, serious mental illness, diabetes, BMI, and family history of prostate cancer. The risk equation explained 70.4% (95% CI = 69.2 to 71.6) of the variation in time to diagnosis of prostate cancer (*R*^2^) (D statistic 3.15, 95% CI = 3.06 to 3.25; Harrell's C-index 0.917, 95% CI = 0.915 to 0.919). Two-step approach had higher sensitivity than a fixed PSA threshold at identifying prostate cancer cases (identifying 68.2% versus 43.9% of cases), high-grade cancers (49.2% versus 40.3%), and deaths (67.0% versus 31.5%).

**Conclusion:**

The risk equation provided valid measures of absolute risk and had higher sensitivity for incident prostate cancer, high-grade cancers, and prostate cancer mortality than a simple approach based on age and PSA threshold.

## INTRODUCTION

Prostate cancer affects an estimated 1 million men worldwide, with >300 000 dying from the disease each year.^1^ Prostate specific antigen (PSA) is a biomarker that is widely used to help detect prostate cancer before symptoms develop or at an early stage. Diagnosing prostate cancer early can potentially identify tumours when they are at an early stage and intervention might improve treatment options and survival; however, studies have suggested that PSA alone has poor senstivity to determine the presence of prostate cancer for any risk stratification category.^2^

A recent meta-analysis concluded that, although screening may result in a small absolute benefit in disease-specific mortality at 10 years, it does not improve overall mortality.^3^ A European trial reported a 27% reduction in prostate cancer mortality attributable to PSA testing at 13 years.^4^ Two other trials, in the US and UK, showed no overall mortality benefit,^5^^,^^6^ although the results might be partially explained by low adherence rates and contamination of the control group.^7^^,^^8^ UK guidelines recommend against systematic prostate cancer screening, instead allowing men aged ≥50 years to request screening on demand.^2^ US guidelines recommend *‘For men aged 55 to 69 years, the decision to undergo periodic prostate-specific antigen (PSA)–based screening for prostate cancer should be an individual one. Before deciding whether to be screened, men should have an opportunity to discuss the potential benefits and harms of screening with their clinician and to incorporate their values and preferences in the decision’*;^9^ however, the tools to achieve this are largely unavailable in both the US and UK, and such shared decision making is seldom undertaken.^8^ A recent *BMJ* rapid review, which summarised all the available evidence on prostate cancer screening with PSA tests, highlighted the need for research to test risk-stratified approaches.^8^

In other clinical areas, such as the prevention of cardiovascular disease, guidelines have evolved from clinical decisions made solely on thresholds of cholesterol, to those made according to absolute risk incorporating other risk factors.^10^^,^^11^ As highlighted recently by Tikkinen *et al*, a similar risk-stratified approach could provide an effective mechanism to improve decision making for doctors and patients by providing realistic estimates of absolute risk of prostate cancer incorporating age, ethnic group, family history, and other risk factors.^8^ This could also reduce unnecessary referrals as it could be applied before undertaking further investigations, such as magnetic resonance imaging (MRI) or biopsies.^7^^,^^12^ A systematic review identified several studies deriving risk equations for predicting absolute risk of prostate cancer incorporating PSA, although the sample sizes were small and not representative of primary care; the populations studied were predominantly White, discrimination was limited, and calibration poorly reported.^13^ Existing calculators have been designed to predict risk of a current diagnosis of prostate cancer, rather than the future risk of developing prostate cancer and/or clinically significant disease over a 10-year period.^14^^–^16

**Table:** How this fits in

Earlier diagnosis of prostate cancer could potentially identify tumours at a stage when interventions could help improve treatment options and survival rates. A new equation to predict the absolute risk of prostate cancer in asymptomatic men with prostate specific antigen (PSA) tests has been developed by the authors and validated externally. The risk equation provides a valid measure of absolute risk of prostate cancer, which is more efficient at identifying incident cases of prostate cancer, high-grade cancers, and prostate cancer deaths than an approach based on a simple PSA threshold. The prostate cancer risk model has the potential to prioritise patients in primary care for further investigation, including imaging by multiparametric magnetic resonance imaging.

Currently, the decision in most primary care practices to refer men who are asymptomatic is based on binary PSA thresholds, although this can lead to too many false-negative and false-positive results.^17^ Furthermore, a binary threshold does not give any indication for the patient as to their absolute risk of developing prostate cancer and/or clinically significant disease requiring immediate intervention. As the diagnostic pathway has evolved considerably — at least in the UK and Europe — PSA level alone no longer triggers prostate biopsy; this is now preceded by a multiparametric MRI (mpMRI) scan. However, mpMRI misses approximately 15% of important prostate cancers (Gleeson grade 2–5) and is difficult to interpret in younger men.^18^

The authors aimed to develop and determine the additional predictive utility of a new algorithm to predict risk of prostate cancer for use in primary care in men who are asymptomatic. The intended use is to provide a better evidence base for the GP and patient to improve decision making regarding the most appropriate action, for example, reassurance, repetition of PSA test, referral for MRI, regular monitoring, referral to a urologist, or use of preventative interventions should any become available.

## METHOD

### Study design, data sources, and sample

The authors undertook a large open cohort study of men registered with 1503 practices contributing to the QResearch database (version 43), which is the largest and most representative GP research database in the UK.^19^ Three-quarters of practices were randomly allocated to the derivation dataset (validation cohort A) and the remaining quarter to a validation dataset. A second validation cohort (validation cohort B) of men registered with general practices contributing to the Clinical Practice Research Datalink (CPRD GOLD) was also identified.

The cohorts included men aged 25–84 years who were registered with practices in the study period (1 January 1998 to 31 March 2018 for QResearch and 1 January 1998 to 31 March 2015 for CPRD) and had at least one PSA test result. Men with a previous diagnosis of prostate cancer were excluded at baseline and, as the aim was to quantify risk in men who were asymptomatic, also excluded were those with recorded evidence of lower urinary tract symptoms, including urinary retention, urinary frequency, nocturia, erectile dysfunction, haematuria, and haematospermia in the 28 days prior to a PSA test — these men were unlikely to be having PSA tests for screening purposes.

An initial entry date to the cohort was determined for each patient, which was the latest of the following:

- 25th birthday;
- date of registration with the practice plus 1 year;
- date on which the practice computer system was installed plus 1 year; or
- the beginning of the study period (1 January 1998).

The date of the first PSA test during the study period after the individual’s initial entry date was then determined; this date was used as the study entry date for the main analysis. Patients were followed up until the earliest of the following dates:

- date of diagnosis of prostate cancer;
- death;
- de-registration with the practice; or
- last upload of computerised data and the study end date (31 March 2018 for QResearch or 31 March 2015 for CPRD GOLD).

All relevant patients on the database were used to maximise the power and generalisability of the results.

### Outcomes

The primary outcome measure was incident diagnosis of prostate cancer during follow-up, as recorded on the general practice computer records or the linked Hospital Episodes Statistics (HES) database, mortality, or cancer registry data (where available). For mortality, men were included as having the primary outcome where prostate cancer was recorded as the main cause of death. The earliest recorded date of prostate cancer on any of these data sources was used as the outcome date.

Secondary outcomes were mortality due to prostate cancer and high-grade prostate cancer, as determined by the Gleason score in which ‘high grade’ constituted a recorded combined score of 7 (4+3), 8, 9, or 10 (Gleason grade group 3, 4, or 5).^20^

### Predictor variables

The selected variables were those previously found to be predictive of prostate cancer (age, self-assigned ethnicity, material deprivation [Townsend score], body mass index [BMI], smoking status, type 1 and type 2 diabetes, serious mental illness, and family history of prostate cancer)^21^ and recorded in patients’ primary care electronic records, as well as PSA levels. The latest information recorded in the GP record on or before the study entry date (that is, the date of the individual’s first PSA test) was used.

### Derivation and validation of the models

A risk prediction equation for prostate cancer diagnosis was developed and validated using established methods.^22^^–^24 The initial analysis was based on patients with complete data. Multiple imputation with chained equations was then used to replace missing values for BMI and smoking status for the main analyses.^25^^–^27 Cox’s proportional hazards models were used to estimate the coefficients for each predictor variable, with Rubin’s rules^28^ used to combine the results across the five imputed datasets.

Fractional polynomials^29^ were used to model non-linear risk relationships with continuous variables (age, BMI, and PSA level). Interactions between predictor variables and age were examined and significant interactions included. The regression coefficients from the final risk equation were used as weights, which were combined with non-parametric estimates of the baseline survivor function^30^ evaluated for each year up to 15 years to derive risk equations.^31^ This enabled risk estimates to be derived for each year of follow-up, with a specific focus on 10-year risk estimates.

### Validation of the model

Multiple imputation was used in both validation cohorts to replace missing values for BMI and smoking status. The final risk equation was then applied to both validation cohorts and measures of discrimination were calculated. As in previous studies,^32^ D statistics,^33^ *R*^2^^,^^34^ and Harrell’s C-statistic evaluated at 10 years were calculated. Calibration was assessed by comparing the mean predicted risks at 10 years with the observed risks, by tenth of predicted risk. Calibration slopes were also calculated, along with discrimination measures for the secondary outcomes of prostate cancer mortality and high-grade cancer.

### Risk-stratified approach

**Figure 1.**
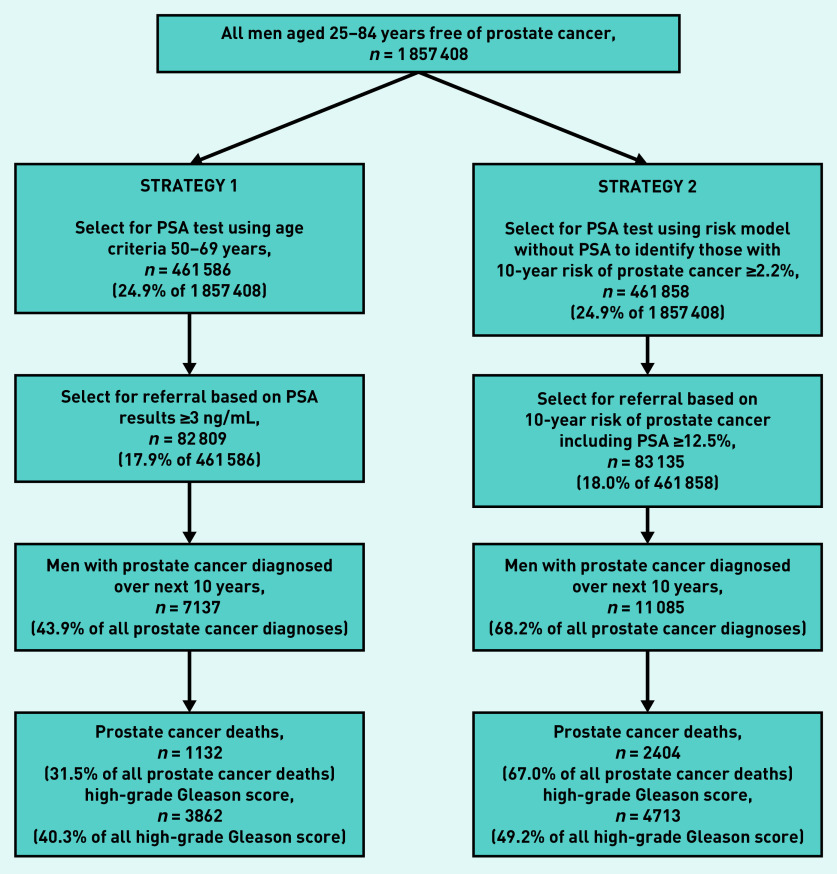
*Comparison of two strategies for identifying men at high risk of prostate cancer using the QResearch validation cohort. PSA = prostate specific antigen.*

To compare performance of the new risk-prediction tool with current UK recommendations,^2^ the sensitivity values for two different strategies for classifying men as high risk of prostate cancer were calculated (Figure 1). The number and proportion of all cases of diagnosed prostate cancer that would be identified over 10 years in the resulting high-risk groups (sensitivity) were then ascertained. The proportion of total prostate cancer deaths and the proportion of high-grade cancer cases identified by each strategy were also calculated.

Stata (version 16) was used for all analyses, and the TRIPOD statement for reporting^35^ was adhered to.

## RESULTS

### Study population and incidence rates

**Figure 2.**
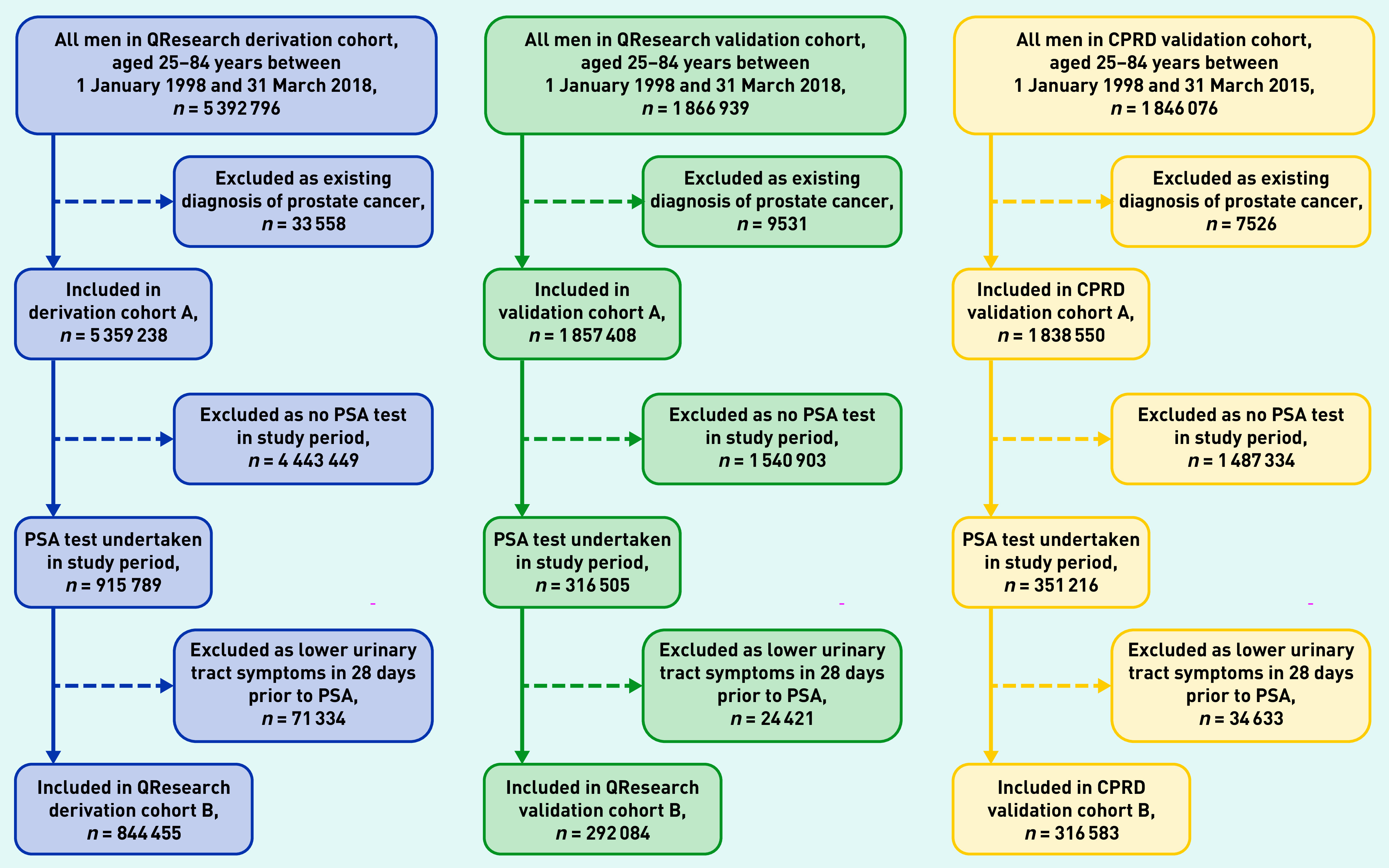
*Patient selection process for QResearch derivation, QResearch validation, and CPRD GOLD validation cohorts. CPRD GOLD = Clinical Practice Research Datalink Global Initiative for Chronic Obstructive Lung Disease. PSA = prostate specific antigen.*

Overall, 1457 QResearch practices (96.9%) were included. Of these, 1098 were randomly assigned to the derivation cohort with the remainder (*n* = 359) assigned to a validation cohort. There were 357 practices in the CPRD GOLD validation cohort. Figure 2 shows the flow of patients resulting in 844 455 men in the QResearch derivation cohort, 292 084 in the QResearch validation cohort, and 316 583 in the CPRD GOLD validation cohort.

Table 1 shows the baseline characteristics of men in the derivation cohort and both validation cohorts. In the derivation cohort, the median age was 57 years. Supplementary Table S1 shows the crude incidence rates for prostate cancer in the QResearch derivation and validation cohorts. There were 40 821 men diagnosed with prostate cancer in the QResearch derivation cohort; of these, 3246 (8.0%) died due to prostate cancer, 11 210 (27.5%) had a high-grade Gleason score, 14 851 (36.4%) had a low-grade Gleason score, and 14 760 (36.2%) did not have a Gleason score recorded. The distribution was similar in both validation cohorts (data not shown).

**Table 1.** Baseline characteristics of men aged 25–84 years and free of prostate cancer and recent urinary symptoms at baseline

**Characteristic**	**QResearch derivation cohort, *n* (%)^a^**	**QResearch validation cohort, *n* (%)^a^**	**CPRD validation cohort, *n* (%)^a^**
Total number of men, *n*	844 455	292 084	316 583

Median age, years (IQR)	57 (48–67)	57 (48–67)	58 (49–67)

Level of deprivation, mean Townsend score (SD)	−0.5 (3.1)	−0.4 (3.1)	−1.2 (3.0)

**Age group, years**			
25–49	244 480 (29.0)	83 294 (28.5)	85 087 (26.9)
50–59	225 655 (26.7)	79 029 (27.1)	89 633 (28.3)
60–69	211 355 (25.0)	72 989 (25.0)	81 253 (25.7)
70–84	162 965 (19.3)	56 772 (19.4)	60 610 (19.1)

**Ethnic group**			
Ethnicity recorded	661 354 (78.3)	228 664 (78.3)	155 947 (49.3)
White/not recorded	763 692 (90.4)	264 163 (90.4)	305 087 (96.4)
Indian	15 883 (1.9)	5428 (1.9)	2693 (0.9)
Pakistani	9501 (1.1)	3087 (1.1)	1012 (0.3)
Bangladeshi	4875 (0.6)	2003 (0.7)	254 (0.1)
Other Asian	8388 (1.0)	2642 (0.9)	1311 (0.4)
Black Caribbean	13 198 (1.6)	4354 (1.5)	1644 (0.5)
Black African	12 631 (1.5)	4704 (1.6)	1750 (0.6)
Chinese	1968 (0.2)	667 (0.2)	293 (0.1)
Other ethnic group	14 319 (1.7)	5036 (1.7)	2539 (0.8)

**Smoking status**			
Smoking status recorded	839 482 (99.4)	290 479 (99.5)	314 742 (99.4)
Non-smoker	421 809 (50.0)	144 973 (49.6)	132 363 (41.8)
Ex-smoker	250 843 (29.7)	86 556 (29.6)	78 345 (24.7)
Light smoker (1–9/day)	96 515 (11.4)	34 647 (11.9)	51 075 (16.1)
Moderate smoker (10–19/day)	36 412 (4.3)	12 709 (4.4)	30 271 (9.6)
Heavy smoker (≥20/day)	33 903 (4.0)	11 594 (4.0)	22 688 (7.2)

**Medical history**			
Median PSA level, score (IQR)	1.18 (1.82)	1.16 (1.76)	1.22 (2.09)
BMI recorded	672 319 (79.6)	234 612 (80.3)	237 333 (75.0)
Mean BMI, kg/m^2^ (SD)	27.2 (4.4)	27.2 (4.4)	26.7 (4.0)
Family history of prostate cancer	8881 (1.1)	2884 (1.0)	1999 (0.6)
Serious mental illness	6475 (0.8)	2386 (0.8)	1946 (0.6)
Type 1 diabetes	2652 (0.3)	890 (0.3)	849 (0.3)
Type 2 diabetes	65 406 (7.7)	23 070 (7.9)	18 512 (5.8)

a *Unless otherwise stated. BMI = body mass index. CPRD = Clinical Practice Research Datalink. IQR = interquartile range. PSA = prostate specific antigen. SD = standard deviation.*

### Predictor variables

Table 2 shows hazard ratios (HRs) for men for both the complete case analysis and the multiply imputed data. The final equation included PSA level, age, deprivation score, ethnicity, BMI, smoking status, family history of prostate cancer, serious mental illness, and type 1 and type 2 diabetes. Increasing deprivation was associated with lower risk of prostate cancer. Black Carribean and Black African men had increased adjusted hazard ratios compared with the White/not recorded group. There were significant interactions between age and family history of prostate cancer, and between age and PSA levels; the adjusted HRs for the fractional polynomial terms for age, BMI, and PSA, as well as interaction terms, are shown in Supplementary Figures S1a–S1e.

**Table 2.** Adjusted hazard ratios (95% CI) for prostate cancer diagnosis for the complete case analysis (*n* = 661 354) and analysis based on multiply imputed data (*n* = 844 455 with five imputed datasets)^a^

**Variable**	**Complete case analysis**		**Imputed data**
	
	**Unadjusted HR (95% CI)**	**Adjusted HR (95% CI)**	**Adjusted HR (95% CI)**
Deprivation score five-unit increase^b^	0.83 (0.82 to 0.85)	0.91 (0.89 to 0.93)	0.91 (0.90 to 0.93)

**Ethnic group**			
White/not recorded	1.00	1.00	1.00
Indian	0.40 (0.36 to 0.45)	0.67 (0.59 to 0.75)	0.67 (0.60 to 0.75)
Pakistani	0.29 (0.24 to 0.35)	0.54 (0.45 to 0.64)	0.54 (0.46 to 0.65)
Bangladeshi	0.16 (0.12 to 0.23)	0.46 (0.33 to 0.65)	0.47 (0.33 to 0.66)
Other Asian	0.33 (0.29 to 0.40)	0.59 (0.50 to 0.71)	0.60 (0.50 to 0.72)
Black Caribbean	1.54 (1.44 to 1.65)	1.56 (1.46 to 1.67)	1.56 (1.46 to 1.67)
Black African	0.80 (0.73 to 0.88)	1.13 (1.02 to 1.25)	1.14 (1.04 to 1.26)
Chinese	0.50 (0.37 to 0.67)	0.54 (0.40 to 0.72)	0.55 (0.41 to 0.73)
Other ethnic group	0.74 (0.68 to 0.82)	1.09 (0.99 to 1.20)	1.10 (1.00 to 1.21)

**Smoking status**			
Non-smoker	1.00	1.00	1.00
Ex-smoker	1.02 (1.00 to 1.05)	1.01 (0.98 to 1.03)	1.00 (0.98 to 1.03)
Light smoker	0.86 (0.83 to 0.89)	0.97 (0.94 to 1.01)	0.98 (0.95 to 1.02)
Moderate smoker	0.77 (0.72 to 0.82)	0.93 (0.88 to 0.99)	0.93 (0.88 to 0.99)
Heavy smoker	0.74 (0.69 to 0.79)	0.94 (0.88 to 1.00)	0.95 (0.90 to 1.01)

**Medical history**			
Family history of prostate cancer^c^	1.47 (1.34 to 1.61)	1.73 (1.55 to 1.92)	1.83 (1.66 to 2.02)
Serious mental Illness^d^	0.52 (0.44 to 0.63)	0.67 (0.56 to 0.80)	0.67 (0.57 to 0.79)
No diabetes	1.00	1.00	1.00
Type 1 diabetes	0.35 (0.25 to 0.49)	0.74 (0.53 to 1.04)	0.78 (0.58 to 1.05)
Type 2 diabetes	0.78 (0.75 to 0.82)	0.90 (0.86 to 0.95)	0.90 (0.86 to 0.94)

a *Model also includes fractional polynomial terms for age (age^−0.5^, age^−0.5^ln[age]) and BMI [BMI ^−1^, BMI ^−0.5^], and PSA (PSA^−1^, PSA^−0.5^) with interaction terms between age terms and family history, and between age and PSA terms.*

b *Increasing Townsend scores indicate increasing levels of deprivation.*

c *Interaction with age; HR evaluated at mean age.*

d *Compared with patients without this characteristic. HR = hazard ratio.*

### Validation

The model had high levels of explained variation and discrimination in both validation cohorts (Table 3). In the QResearch validation cohort, the model explained 70.4% of the variation in time to diagnosis of prostate cancer (*R*^2^), the D statistic was 3.15, and Harrell’s C-statistic was 0.917. For prostate cancer death, *R*^2^ was 66.2%, the D statistic was 2.86, and Harrell’s C-statistic was 0.907. For high-grade cancer, these values were as follows: *R*^2^ = 66.7%, D statistic = 2.90, and Harrell’s C-statistic = 0.935. The corresponding figures in the CPRD validation cohort are shown in Table 3.

**Table 3.** Performance of the risk model to predict prostate cancer time to diagnosis, prostate cancer death, and high-grade prostate cancer in the QResearch validation and CPRD validation cohorts

	**QResearch validation cohort**		**CPRD validation cohort**	
	
	**Complete data (*n*= 188 013), estimate (95% CI)**	**Imputed data (*n*= 292 084), estimate (95% CI)**	**Complete data (*n*= 120 869), estimate (95% CI)**	**Imputed data (*n*= 316 583), estimate (95% CI)**
**Prostate cancer time to diagnosis**				
Harrell’s C-statistic	0.920 (0.917 to 0.923)	0.917 (0.915 to 0.919)	0.922 (0.919 to 0.925)	0.916 (0.914 to 0.918)
D statistic	2.71 (2.67 to 2.75)	3.15 (3.06 to 3.25)	2.83 (2.78 to 2.87)	2.82 (2.79 to 2.85)
*R*^2^	63.7 (62.8 to 64.5)	70.4 (69.2 to 71.6)	65.6 (64.5 to 66.7)	65.5 (65.1 to 65.9)

**Prostate cancer death**				
Harrell’s C-statistic	0.909 (0.895 to 0.923)	0.907 (0.897 to 0.917)	0.901 (0.865 to 0.937)	0.906 (0.894 to 0.918)
D statistic	2.84 (2.69 to 2.99)	2.86 (2.76 to 2.97)	3.10 (2.78 to 3.42)	3.16 (3.04 to 3.28)
*R*^2^	65.9 (63.4 to 68.3)	66.2 (64.6 to 67.8)	69.6 (64.8 to 73.6)	69.4 (68.8 to 72.0)

**High-grade prostate cancer**				
Harrell’s C-statistic	0.934 (0.930 to 0.939)	0.935 (0.932 to 0.938)	—	—
D statistic	2.88 (2.82 to 2.95)	2.90 (2.85 to 2.95)	—	—
*R*^2^	66.5 (65.3 to 67.7)	66.7 (65.9 to 67.6)	—	—

Supplementary Figure S2 shows how discrimination varied across practices in the QResearch and CPRD validation cohorts.

The calibration slope was 1.03 (95% CI = 1.02 to 1.04) for the CPRD validation cohort and 0.99 (95% CI = 0.98 to 1.01) for the QResearch validation cohort. Supplementary Figure S3 shows that the equation is well calibrated overall and in each subgroup.

Supplementary Table S2 shows the sensitivity, specificity, and observed 10-year risk based on tenths of predicted 10-year risk of prostate cancer diagnosis in the QResearch validation cohort; as an example, in the top tenth of risk (that is, men with a 10-year predicted risk of ≥20.1%), the sensitivity was 65.5%, specificity 92.6%, and observed risk was 36.7%.

### Risk stratification and clinical use

Figure 1 compares two strategies for identifying men at high risk of prostate cancer using the QResearch validation cohort. The two-step approach (strategy 2) had higher sensitivity than the fixed PSA threshold (strategy 1) at identifying prostate cancer cases (identified 68.2% versus 43.9% of cases), high-grade cancers (49.2% versus 40.3%), and deaths (67.0% versus 31.5%).

Supplementary Figure S4 shows the web calculator with clinical examples to demonstrate how the risk model could be used in a consultation. A 35-year-old Black Caribbean man with a PSA level of 3 ng/mL without a family history of prostate cancer has a 6.7% risk of developing prostate cancer over the next 10 years. With a family history of prostate cancer, his 10-year risk of prostate cancer would be 38.2%.

## DISCUSSION

### Summary

The QResearch database was used to develop the prostate cancer risk model in men who were asymptomatic. The model was then externally validated using two separate validation cohorts. The analyses included 1.45 million men from UK primary care over a 20-year period. The results show that the risk equation provides a valid measure of absolute risk and is more efficient at identifying incident cases of prostate cancer, high-grade cancers, and prostate cancer deaths than an approach based on a PSA threshold. A publicly available calculator has been developed to implement the algorithm that can be used to communicate levels of risk to patients to aid shared decision making.

### Strengths and limitations

A key strength of the study is the use of a large primary care database making it substantially larger and more representative of the general population than previous studies. Other key strengths include: duration of follow-up and lack of selection, recall, and responder bias. UK general practices have good levels of accuracy and completeness in recording clinical diagnoses and investigations,^36^ and this will allow the risk equation to be updated as data changes over time. The methods used to derive and validate these models are established approaches as used for other risk-prediction equations derived from the QResearch database.^37^^–^39

Limitations of the study include the lack of formal adjudication of diagnoses of prostate cancer, although the authors used multiple linked data sources. In addition, there was a potential under-ascertainment of family history of prostate cancer or high-grade Gleason scores, as not all patients had recorded values. There may also have been some patients in the study cohorts who had undiagnosed prostate cancer. Nonetheless, these limitations are likely to also occur in the clinical setting, where the results are likely to be used and, hence, have a face validity.

### Comparison with existing literature

The HRs for established predictors were similar to those reported elsewhere: family history of prostate cancer was associated with a higher risk of prostate cancer, as in other studies,^40^ and Black African and Black Caribbean men had significantly higher risks compared with White men.^41^ Serious mental illness was associated with a lower risk of prostate cancer compared with not having serious mental illness, as reported elsewhere.^42^ Diabetes was also associated with a lower risk of prostate cancer compared with not having diabetes, in line with previous studies;^43^^,^^44^ this has been postulated as being either a detection bias or a possible protective association of diabetes medication.^45^

The risk prediction tool outlined in the study presented here improved on the Prostate Cancer Prevention Trial^14^ and European Randomized study of Screening for Prostate Cancer^15^^,^^16^ calculators as it:

- was developed from a large, representative primary care population including almost 1.45 million men, compared with trial populations of several thousand men already selected for biopsy;
- included established risk factors;
- can be used to predict short-term and longer-term absolute risks;
- used existing information from electronic health records and, as such, can be easily implemented in a primary care setting;
- can be updated in line with changes in the population, clinical data, and clinical practice; and
- has been externally validated.

In addition, the equation has been published for transparency.

### Implications for research and practice

The authors have developed and externally validated a risk equation to quantify 10-year risk of prostate cancer in men who are asymptomatic and undergoing a PSA test. This warrants further research to assess utility of the model to prioritise men in primary care for further investigation, such as mpMRI. Further research is needed to assess how best to implement the algorithm, and evaluate cost-effectiveness and the impact on prostate cancer diagnosis and subsequent survival.
